# A nationwide collapse of a priority grassland bird related to livestock conversion and intensification

**DOI:** 10.1038/s41598-023-36751-8

**Published:** 2023-06-29

**Authors:** João Paulo Silva, Ana Teresa Marques, Carlos Carrapato, Rui Machado, Rita Alcazar, Ana Delgado, Carlos Godinho, Gonçalo Elias, João Gameiro

**Affiliations:** 1 CIBIO, Centro de Investigação em Biodiversidade e Recursos Genéticos, InBIO Laboratório Associado, Universidade do Porto, Campus de Vairão, 4485-661 Vairão, Portugal; 2 BIOPOLIS Program in Genomics, Biodiversity and Land Planning, CIBIO, Campus de Vairão, 4485-661 Vairão, Portugal; 3 Estação Biológica de Mértola, 7750-329 Mértola, Portugal; 4 CIBIO, Centro de Investigação em Biodiversidade e Recursos Genéticos, InBIO Laboratório Associado, Instituto Superior de Agronomia, Universidade de Lisboa, 1349-017 Lisbon, Portugal; 5 ICNF/PNVG-Instituto de Conservação da Natureza e Florestas, Parque Natural do Vale do Guadiana, 750-350 Mértola, Portugal; 6 SPEA-Sociedade Portuguesa Para o Estudo das Aves, Av. Alm. Gago Coutinho 46A, 1700-031 Lisbon, Portugal; 7 LPN-Liga Para a Proteção da Natureza, Centro de Educação Ambiental de Vale Gonçalinho, 7780-909 Castro Verde, Portugal; 8 Rua Gil Vicente, 5, 7780 094 Castro Verde, Portugal; 9 MED-Instituto Mediterrâneo Para a Agricultura, Ambiente e Desenvolvimento, LabOr-Laboratório de Ornitologia, Universidade de Évora, Polo da Mitra, 7002-774 Évora, Portugal; 10 Aves de Portugal, Rua de São Pedro, 44, 7320-163 Castelo de Vide, Portugal

**Keywords:** Agroecology, Conservation biology, Grassland ecology

## Abstract

Grassland birds are among the most threatened and fastest declining terrestrial vertebrate species in Europe, principally due to agricultural intensification and transformation. The little bustard is a priority grassland bird under the European Directive (2009/147/CE) that led to the classification of a network of Special Protected Areas (SPAs) in Portugal. A third national survey carried out in 2022 reveals a worsening of an ongoing population collapse at a national scale. The population declined by 77% and 56% compared to the previous surveys in 2006 and 2016, respectively. We found that the little bustard has greatly disappeared outside SPAs, while the remaining breeding population concentrated within the protected area network is showing a steep decline at a rate of 9% a year. This decline is now twice as fast when compared to the period 2006–2016. Analysis of the variation of the breeding densities between 2006 and 2022 at 49 survey sites revealed that those that initially had higher bustard densities and shifted toward a higher proportion of cattle among the total stocking rate experienced steeper declines. Areas where the density of roads increased also experienced declines over the course of the study period. Agricultural areas converted to or dominated by beef production likely relate to low breeding success and mortality of nesting females in fodder crops. Still, major habitat conversion outside SPAs to permanent crops led to overall habitat destruction, which contributed to the species decline and range contraction. Other threats are likely acting synergistically such as fragmentation, climate change and anthropogenic mortality. The extinction of the little bustard in Portugal is expected in the short term if no conservation actions are put in place.

## Introduction

Grassland birds are among the most endangered terrestrial vertebrates in Europe, threatened by habitat transformation mainly by agricultural and pastoral intensification^1–3^. The Iberian Peninsula is an important stronghold for many grassland birds in Europe, many of which with unfavourable conservation status. The little bustard (*Tetrax tetrax*) is one such species, classified globally as Near Threatened and Vulnerable both in Europe and Portugal^4,5^. It is also a priority species for conservation under the European Bird Directive (2009/147/CE), and as a result, a vast network of Special Protection Areas (SPAs) was designated with the goal of maintaining or enhancing its conservation status. At the turn of the millennium, Portugal's little bustard population showed indicators of good conservation status^6,7^. The first national survey, conducted between 2003 and 2006, revealed widespread high breeding densities that, in some regions, corresponded to the highest breeding densities ever recorded for the species^8^. In just 10 years the breeding population crashed, with a nationwide decline of approximately 50%^7^, with greater rates of decline seen in regions where the proportion of cattle in the total stocking rate was higher^9^. Portugal's agricultural policy changed particularly over the last two decades, replacing extensive dry cereal cultivation with one centred on permanent pastures for beef production that tended to be intensified with a growing number of grazers^10^. Due to shorter vegetation swards, the quality of the breeding habitat was impacted, making it unsuitable for breeding^11^.

The Common Agricultural Policy (CAP), which was designed to increase food self-sufficiency, has been the primary cause of habitat loss or degradation for farmland birds in Western Europe^12^. When the CAP funds in Portugal stopped supporting cereal farming in 2005, they were largely replaced by subsidies to encourage and intensify cattle grazing, which became the main driver of habitat conversion. On the other hand, substantial cereal areas outside SPAs were converted to irrigation and replaced mostly into permanent crops, such as olive groves, orchards, and vineyards, which resulted in a complete loss of breeding habitat for the species.

The little bustard in Iberia is well adapted to low-intensity cereal farming and extensive pastures to breed^8^. It exhibits an exploded lekking breeding system, where territorial males display in a somewhat congregated manner that are then visited by females to mate^13^. Adults primarily eat green plants, whereas chicks feed exclusively on arthropods for the first two to three weeks of their lives^14^. Breeding population estimates are typically based on adult male densities because females are too inconspicuous to be detected in workable numbers^15^.

Previous surveys show that population declines in Portugal were also observed at sites where the density of power lines was greater^9^. Powerlines are a known and significant source of adult mortality^16–18^ and are avoided during the breeding season, resulting in lower densities next to these infrastructures^19^.

The purpose of this study is to examine the trends of the little bustard in Portugal using data from the most recent survey, which was conducted during the breeding season of 2022, and analyse the main factors explaining density variation between surveys.

## Methods

### Little bustard surveys

**Figure 1:**
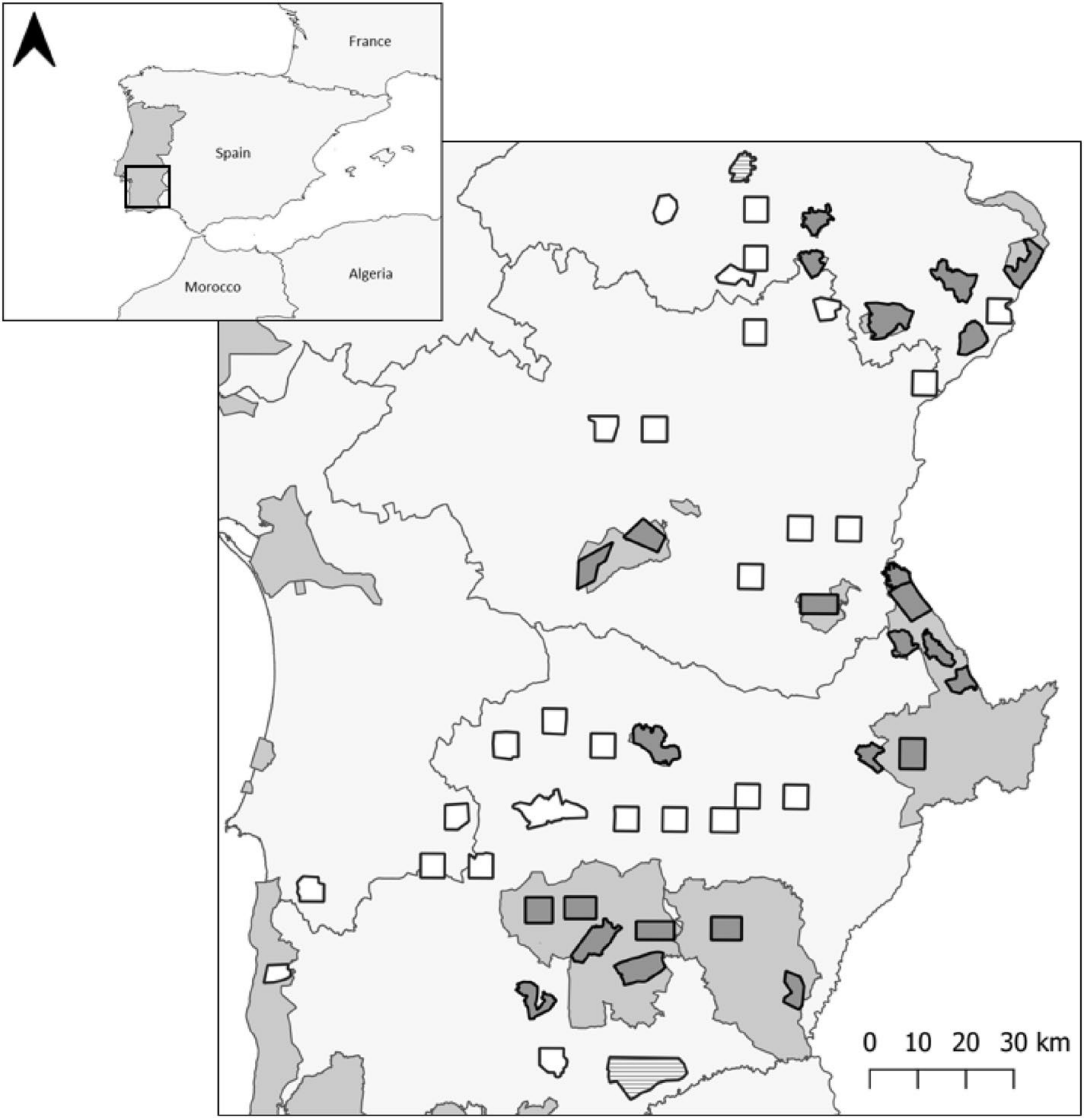
Location of the 55 areas surveyed in 2022 in Alentejo, Portugal. Darker, white and striped polygons correspond to areas surveyed within SPAs, outside SPAs, and IBAs, respectively. Special Protection Areas and Important Bird Areas are represented in intermediate grey.

The little bustard surveys took place in the region of Alentejo, Portugal, which concentrates over 95% of the breeding population in Portugal^20^, following a standardized protocol based on the estimation of male densities^6^. Surveys were conducted in three different periods: between 2003 and 2006, in 2016, and in 2022—in predetermined areas. The first survey was carried out by experienced ornithologists hired specifically for the project, while the following surveys were done by volunteers, CIBIO, the ICNF administration, and non-governmental organizations. Preparation sessions ensured standardized data collection for those who had no prior experience surveying the species. The first survey was based on a network of 81 areas, 51 of which were repeated in 2016^7^. In 2022, a total of 55 areas were surveyed in (Fig. 1): 25 located within 13 SPAs (mean = 3025 ha, min = 1715 ha, max = 4718 ha), 2 within Important Bird Areas (IBAs) (mean = 5652 ha, min = 2000 ha, max = 9303 ha), and 28 areas located at random agricultural areas outside SPAs and IBAs (mean = 2564 ha, min = 1657 ha, max = 5926 ha)^6,7^. Of these, 49 areas coincided with areas surveyed across all three censuses. The protocol for estimating male densities in each survey area consisted of a network of survey points previously established along dirt tracks and spaced 600 m apart from one another, located away from disturbance elements like paved roads or inhabited houses. From the 8th to the 30th of April, points were surveyed early in the morning or late in the day (7 h-10 h and 17 h-19 h), for a duration of five minutes each, and every little bustard male within a 250 m radius was recorded. Points were repeated between surveys at the exact same locations. For the survey of 2016, a random stratified selection of the survey areas of 2003–2006 outside SPAs were carried out and in 2022 replicated. Overall, a total of 2 326, 1 441 and 1 493 points were surveyed for the first, second, and third national surveys, respectively.

For each survey area, the number of males found within the 250 m buffer of the sampled points was used to estimate the mean male density (and 95% confidence intervals). Mean density determined from the survey points was then extrapolated to the entire potential habitat area within SPAs, or to the entire survey area outside SPAs. For each survey, the overall national population estimate thus resulted in the sum of the estimates of each area surveyed that year. The mean density for large SPAs with multiple sampled areas was determined by averaging the densities of all the areas. We measured the proportional change in estimated population sizes across SPAs and outside areas between each of the three surveys and on an annual basis (by dividing the decline by the number of years between surveys). For more details on the survey protocol, please refer to Moreira et al. (2012) and Silva et al. (2018).

### Habitat availability and quality

We used the approach of Marques et al. (2020) to identify potential drivers of the variation in bustard population density from 2003–2006 to 2022 using the 49 areas sampled concurrently in the three national surveys. To accomplish this, we modelled the variation in absolute density values from 2003–2006 to 2022 and related it to known drivers of species distribution and abundance. The analysis was based on seven variables from three major drivers—breeding habitat availability, livestock management and linear infrastructures—that were collected for the 49 areas (Table 1).

**Table 1:** Description of the predictor variables used to model little bustard density variation in Alentejo, Portugal.

Driver	Variable	Description
Habitat quantity	Habitat	Proportion of the survey area covered with potential breeding habitat: non-irrigated annual crops, permanent pastures and fallow land
Habitat quality: livestock management	#Sheep/ha	Number of sheep per area of pastures and fallow land (LU/ha)
	#Cattle/ha	Number of cattle per area of pastures and fallow land (LU/ha)
	Stocking rate	Density of cattle and sheep livestock units per area of pastures and fallow land (LU/ha)
	Cattle proportion	Proportion of cattle in the stocking rate: #Cattle/ha / (#Cattle/ha + #Sheep/ha)
Habitat quality: infrastructures	Roads	Density of paved roads in each survey area. The length of the structures at the survey area boundaries was divided in half (km/ha)
	Power lines	Density of power lines in each survey area (km/ha)

#### Habitat availability

The proportion of surface covered by suitable breeding habitat for the little bustard was calculated for each sampling area (Table 1). Suitable habitat included the following land uses: permanent pastures, non-irrigated annual crops and fallow lands^6,8,21^, represented in the classes 2.1.1 and 2.3.1 of the land use maps of mainland Portugal for 2007, 2015 and 2018, publicly available on-line at https://snig.dgterritorio.gov.pt/. Land use maps were later refined to match the survey years based on visual inspection of Google Earth (Maps data: Google, Landsat, Copernicus) and on field validation.

#### Habitat quality—livestock management

Agricultural data from the national agrarian census (RGA—*Recenseamento Geral Agrícola*) of 1999, 2009, and 2019 (publicly available from www.ine.pt) were used to characterize livestock densities in our survey areas, with a focus on the region's two main grazers: cattle and sheep. We used four different statistics (Table 1): (1) total number of sheep per area of fodder (i.e., the area covered by permanent pastures, fallow lands and natural meadows); (2) total number of cattle per area of fodder; (3) stocking rate, i.e., the number of livestock units (LU) per area of fodder (stocking rates were calculated according to the following ratio: cattle = 1 LU; adult sheep = 0.15 LU^22^); and (4) the proportion of cattle in the total (cattle + sheep) stocking rate. To obtain an estimate for each sampling area, we used the data from the smallest administrative region in the country (i.e., *Freguesia*) as our unit and applied a weighted mean based on the area occupied by each *Freguesia* in our individual survey areas. Due to the time lag between the little bustard surveys and the available livestock data, we used the mean value between 1999 and 2009 data as a proxy for 2003–2006 census, the mean value of 2009 and 2019 data as a proxy for the 2016 survey and the 2019 data as a proxy for the last survey (2022).

#### Habitat quality—linear infrastructures

In each survey, we collected data on the distribution of paved roads and power lines and calculated their density (m/ha) per study area (Table 1). We identified the main paved roads using data from Open-StreetMap contributors^23^, specifically within one of the following categories: motorway, trunk, primary, and secondary. Using data provided by Portugal's electric companies (REN and EDP), we mapped both the transmission (> 110 kV) and distribution (110 kV) networks for power lines. Data on these linear infrastructures were validated using Google Earth, Bing images, and field checks for each survey period. When roads coincided with the limit of the sampling area, we assumed they had a lower effect than those traversing them. As a result, the length of the roads at the survey areas' boundaries was down-weighted when calculating density by dividing the length in half, similar to the approach used in a previous work^9^.

### Data analysis

We used Generalized Additive Models (GAM) to identify the main drivers influencing the variation in little bustard density over time, thereby accounting for potential non-linear responses^24,25^. The variation in little bustard density across time (survey 2022–survey 2003/2006) in each area was used as the response variable. Predictors included the variables related to habitat availability, livestock management and linear infrastructures for 2022, therefore characterizing the habitat and potential pressures of each area. We also included the predictors’ variation across the same period as the explanatory variables. Furthermore, because the magnitude of the absolute variation in density is constrained by the initial value in the area, the little bustard density in the first survey was also included as a predictor. We used Spearman correlation coefficient and variance inflation factors to check for collinearity between the explanatory variables^25^. Regarding the livestock management predictors, the number of sheep and cattle were both correlated with both the stocking rate and cattle proportion in the stocking rate, so only the latter two predictors were included in the models. For the other predictors, variance inflation factor values (all < 3.0) and pairwise correlations (all |r|< 0.50) were low, so all were used in the analysis.

The modelling procedure involved fitting the full model, followed by backward elimination of non-significant (p > 0.05) variables to find the optimal model^25^. GAMs were fitted with a Gaussian distribution and an identity link function. The optimal smoothing parameter was estimated by restricted maximum likelihood estimation (REML), and a basis dimension (k = 3) was defined to allow some complexity in the functions while avoiding over-fitting the data. The final model adequacy was evaluated by plotting residuals versus fitted values and explanatory variables, and the model fit was evaluated by the proportion of the null deviance explained^25^. Spline correlogram plots with 95% pointwise confidence intervals calculated with 1000 bootstrap resamples were used to check for spatial autocorrelation in model residuals^26^. We assumed that variable selection and parameter estimation were unbiased if there was no significant autocorrelation in model residuals^27^. GAMs were fitted with the package *mgcv*^28^, and correlograms were estimated with *ncf* package^29^, both in R^30^.

## Results

### Population trends

**Figure 2:**
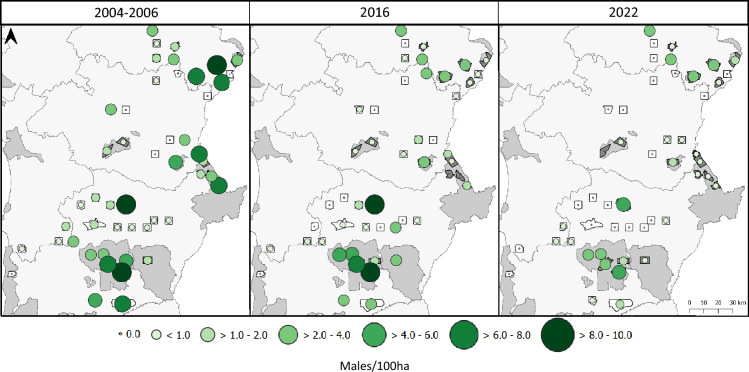
Evolution of the little bustard male density (birds/100 ha) in the 49 areas sampled concurrently in the three surveys. Darker and larger circles correspond to higher male density.

The little bustard survey of 2022 showed a sharp decline of 56% when compared with the 2016 result, in just 6 years, and a negative variation of 77% compared with the first survey between 2003 and 2006 (Table 2, Fig. 2). Outside SPAs and IBAs hardly any breeding males were recorded during this last survey (only ten birds) showing a negative trend of 94% compared to the 2003–2006 estimate. Within SPAs all areas showed a negative trend, and the non-SPA IBA of Alter do Chão was the only surveyed area that showed a positive trend, but irrelevant in the national context since the variation was of only 6 males (Table S1). Overall estimates of breeding males within SPAs show that in the 2022 survey there were 54% fewer breeding males compared to 2003–2006 and less 38% compared to 2016. In summary, now breeding little bustards occur mainly in SPAs and 2 additional IBAs and are mostly absent from the remaining territory. For 2022, the average breeding population was estimated at 3944 breeding males, less 13 475 males than what had been estimated for the first survey 16 years ago (Table 2).

**Table 2:** Results of the three little bustard national surveys carried out in mainland Portugal, representing the estimates (mean, minimum, maximum) of the number of males and variation (difference in mean estimates number and proportion) within and outside Special Protection Areas (SPAs).

	Estimate									Variation					
	2003–2006			2016			2022			2003/2006–2016		2016–2022		2003/2006–2022	
	Mean	Min	Max	Mean	Min	Max	Mean	Min	Max	#	%	#	%	#	%
SPAs	6695	3875	9514	5008	2701	7790	3114	1358	4959	− 1687	25.2	− 1894	− 37.8	− 3581	− 53.5
Non-SPAs	10,724	9199	12,248	3892	2739	5045	830	67	1864	− 6832	63.7	− 3062	− 78.7	− 9894	− 92.3
IBAs	690	525	855	304	122	485	243	67	419	− 386	55.9	− 61	− 20.1	− 447	− 64.8
Non-IBAs	10,034	8674	11,393	3588	2617	4560	587	0	1445	− 6446	64.2	− 3001	− 83.6	− 9447	− 94.1
Total	17,419	13,074	21,762	8900	5540	12,835	3944	1425	6823	− 8519	48.9	− 4956	− 55.7	− 13,475	− 77.4

All areas surveyed in each survey included.

### Habitat trends

**Figure 3:**
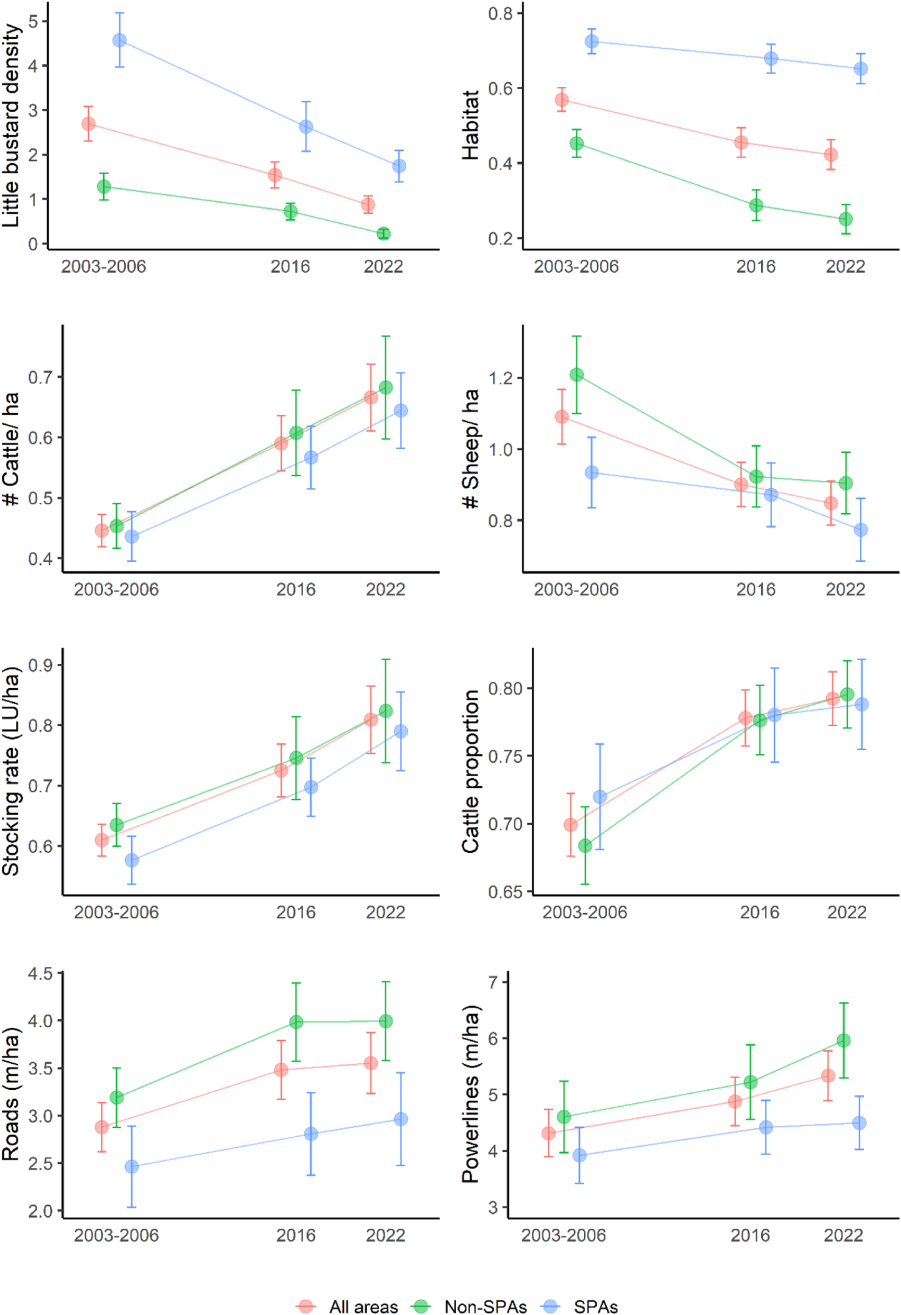
Variation in little bustard density and environmental predictors (mean and standard errors) between the three surveys periods.

The availability of suitable breeding habitat for the little bustard has decreased over time (from an average of 56.9 ± 22.1% in 2003–2006 to 42.3 ± 28.0% in 2022) (Fig. 3), with only 20 areas retaining the original amount of habitat. Although the decline is more pronounced outside SPAs, we also recorded some losses within them, from 72.5 ± 14.8% in 2003–2006 to 65.2 ± 18.4% in 2022.

Livestock management has changed sharply since the first survey (Fig. 3). The number of sheep decreased over time (1.1 ± 0.5 sheep/ha in 2003–2006 and 0.9 ± 0.4 sheep/ha in 2022), while the number of cattle increased (0.5 ± 0.2 cattle/ha in 2003–2006 and 0.7 ± 0.4 cattle/ha in 2022). The transition from sheep to cattle grazing was also accompanied by an increase in the overall stocking rate, which has been steadily increasing since 2003–2006 (0.6 ± 0.2 livestock units in 2003–2006 and 0.8 ± 0.4 livestock units in 2022). As a result, the proportion of cattle in the total stocking rate increased over the study period (69.9 ± 16.3% in 2003–2006 and 79.2 ± 13.9% in 2022). This trend was observed throughout the study area, but grazing pressure was lower within SPAs, with fewer livestock units and a lower stocking rate when compared with the other study areas.

Linear infrastructures have also been increasing since 2003–2006 (roads: 2.9 ± 1.8 km/ha in 2003–2006 to 3.6 ± 2.2 km/ha in 2022; powerlines: 4.3 ± 3.0 km/ha in 2003–2006 to 5.3 ± 3.1 km/ha in 2022) (Fig. 3). Although roads and powerlines were less common within SPAs, the network has been steadily expanding (roads: 2.5 ± 2.0 km/ha in 2003–2006 and 3.0 ± 2.2 in 2022 km/ha; powerlines: 3.9 ± 2.3 km/ha in 2003–2006 and 4.5 ± 2.2 km/ha in 2022).

### Drivers of population variation

**Figure 4:**
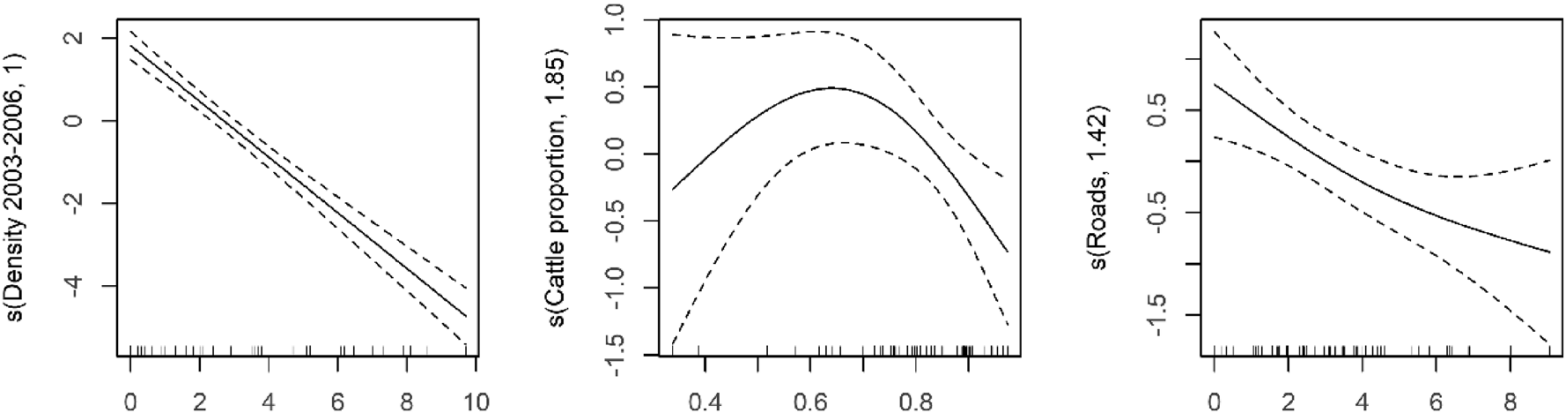
Smoothed curve of the additive effect of the environmental predictors in the GAM model on the variation in little bustard density over time (from the first survey in 2003–2006 to the current survey in 2022). Dashed-lines represent 95% confidence intervals, marks along the x-axis represent a single observation. The y-axis shows the contribution of the fitted centred smooth terms s (names of the predictor, estimated degrees of freedom) to the response variable (variation in little bustard density between surveys).

Larger declines in little bustard density occurred in areas with higher densities of the species in the first survey, a higher proportion of cattle (> 70%) in the stocking rate (during 2022) and a higher density of roads (during 2022) (Table 3 and Fig. 4). The explained deviance was 84%, suggesting that this model has high explanatory power and predictability (Table 3). There was no significant autocorrelation in model residuals (Fig. S1). When analysing the variation of the predictors between 2003–2006 and 2022, no variable was retained in the final model.

**Table 3:** Summary statistics for the GAM modelling the variation in little bustard density over time (survey 2022–survey 2003–2006).

Model coefficients	Estimate	SE	t	edf	F	p-value	Deviance explained
Intercept	− 1.82	0.12	− 15.38			0.000	84%
Density (2003–2006)				1	219.23	0.000	
Cattle proportion				1.85	5.08	0.017	
Roads				1.42	8.32	0.005	

*SE* standard error, *t* T statistics, *edf* estimated degrees of freedom, *F* F statistics.

## Discussion

Here we present the results of the third national survey of the little bustard in Portugal, conducted during the breeding season of 2022. Utilizing the same methodology at the exact same locations as the previous two surveys (in 2003–2006 and in 2016), we reveal an intensification of the declining trend linked with changes in agricultural policy and expansion of infrastructures which, if not reverted, could lead to the extinction of the species in the country in the short term.

### Population trends

The breeding male population declined at an estimated rate of 5% a year between the first and second surveys (a decline of 50% over a ten-year period). With 3944 little bustard males estimated in 2022, the rate of decline has almost doubled in just 6 years and is currently over 9% per year. The decline was steeper outside than within classified areas (SPAs and IBAs), increasing the rate of decline from 6% a year from the first to second surveys to 14% a year from the second to the third survey. While still holding close to 600 breeding males, areas outside SPAs and IBAs lost 94% of the breeding population since 2003–2006. The two areas outside SPAs where the species still subsists are two IBAs where the decline rate actually decreased from 6 to 2% a year. Conversely, all 13 SPAs showed declining trends since 2006 (Table S1), at an average decline above 50%. During 10 years, between the first and second surveys, little bustards within SPAs declined at a rate of 3% per year, which increased markedly to 6% a year in the last 6 years. Overall, the country's population declined steeply by 77% in just 16 years, from a breeding population of roughly 17,500 males in 2006 to fewer than 4,000 males in 2022.

### Drivers of breeding population decline

When we compared the outcomes of our modelling procedure with the previous analysis based on the first two surveys, 2003–2006 and 2016^9^, we found similar findings on the main drivers explaining the variation in little bustard density. Areas with higher bustard densities during the first survey and with higher proportion of cattle within the livestock rate were related to steeper declines. These areas with initial higher breeding densities coincided with well-conserved sites that were subsequently degraded, leading to greater absolute little bustard losses^7^. Greater declines were also associated with a higher density of roads.

Our model most likely better captured the patterns seen within SPAs, where the breeding density variation between surveys was greater, when compared with what was observed outside SPAs. Breeding densities outside of SPAs were low in 2003–2006, despite being widespread and holding a significant portion of the population, which resulted in an overall low variation in breeding density with the overall decline registered in 2022. Nonetheless, the significant loss of breeding habitat observed outside of SPAs (driven by the conversion to permanent crops) is likely to have contributed to the decline, contraction, and isolation of the remaining breeding populations.

The EU’s Common Agricultural Policy (CAP) incentives, over the last two decades, contributed decisively towards a major conversion from dry cereal farming to permanent pastures (mostly for beef production) within the full extent of the little bustard’s distribution in Portugal^8,10^. While the impact of this conversion to little bustards was already identified previously^9^, here we demonstrate that these changes are still ongoing and have even been intensified. The current management of cattle farms for beef production is extremely specialised, simplifying the landscape towards permanent pastures and to a lesser extent fodder crops, mostly hay fields. Additionally, these systems have been intensified with a notable rise in the quantity and intensity of grazing^9,10^, despite the increasing number of severe droughts that greatly reduce plant biomass in pastures. Overgrazing leads to habitat loss or degradation, impacting the vegetation structure, with smaller vegetation swards^11^. The increase in frequency and severity of drought episodes related to climate change^32^, will further impact the vegetation structure and deteriorate the breeding habitat. The consequences of overgrazing may also relate to food availability, since the intensity of grazing has been shown to affect insect abundance^31^. Arthropods, particularly Orthoptera and Coleoptera, are critically important for the development of little bustard chicks^14^, and lack of trophic availability may jeopardise breeding productivity^10^.

On the other hand, hay fields are likely acting as an ecological trap by providing suitable nesting habitat, which is then cut at peak nesting stage, destroying the nests, chicks, and adults, as shown in a previous study^33^. A recent study shows that the breeding populations of the little bustard in Iberia have a skewed sex-ratio towards males at levels which may increase the probability of extinction, supporting the hypothesis that the viability of little bustard populations in Western Europe is threatened by an excess of female mortality^34^. A similar threat was found in France, where the destruction of nests during the mowing of alfalfa fields was identified as one of the main factors contributing to breeding failure and to over 90% population decline of the species between 1980 and the late 1990^35^.

As for the effect of anthropogenic infrastructures, roads were identified as an important predictor explaining the variation in little bustard breeding density. Previous works have found roads create an avoidance effect^36,37^. Powerlines, on the other hand, did not show a relationship with varying densities, contrary to what was found when analysing the period 2003–2016^9^. This is most likely related to the shift in the species distribution, which are now mainly within SPAs, whereas most power lines are concentrated outside SPAs.

### Other factors of decline

The factors identified in this work relate to the decline of little bustard densities, which occurred principally within the context of SPAs. Areas outside SPAs, on the other hand, have been subjected to considerable habitat loss and degradation, a result of major investments in irrigation schemes that have enabled more intensive agriculture principally through permanent crops (olive or almond). Most irrigation schemes are located in areas with more productive soils, which historically coincided with lower breeding densities areas but which were important for the little bustard during the post-breeding season due to their greater availability of food resources, during the hottest and driest season of the year^38^. Consequently, habitat loss and fragmentation of these areas may lead to greater energy expenditure when searching for suitable post-breeding areas (usually outside SPAs), which may also increase bird’s risk of collisions with power lines. Additionally, the intensification of agriculture and increased use of pesticides contributes significantly to the decline of farmland birds, either indirectly limiting habitat and food availability sources or directly through toxic effects that affect bird survival, fitness, or reproduction^39^.

Non-natural mortality is also high and likely contributing to the species’ decline^16^, particularly if we take into account that the species has a very low breeding productivity^34^. Powerlines alone are responsible for an estimated 3.4–3.8% of adult annual mortality, while poaching reaches another 2.4–3%^16^, being particularly vulnerable in non-breeding areas^21^.

### Conservation implications

In 2010 the Iberian breeding population harboured more than half of the world population^40^, but has undergone steeply mostly due to PAC-driven habitat loss and degradation^10,41,42^. The current rate of decline of the little bustard in Portugal is severe and unsustainable. Possible causes of the widespread and rapid decline include a number of threats that are acting synergistically. Permanent pastures for beef production relate to greater losses in breeding density and likely represent an ecological trap, compromising breeding productivity and a potential source of female mortality. The conversion of potential breeding habitat into permanent crops, on the other hand, has resulted in significant habitat loss outside of SPAs, which has caused further population decline and range contraction. Adding to other threats such as non-natural mortality and climate change, the species extinction is inevitable in the short term if no action is put in place. SPAs are now isolated and present a metapopulational structure, which could challenge the bustards to perform greater post-breeding movements towards areas with greater food availability during the post-breeding summer season.

Previous works have shown that the implementation of targeted agri-environmental programs based on the identification of limiting factors can halt population decline or even recover depleted populations^43–45^. In Portugal, several SPAs were designated based on the national and European importance of breeding little bustards’ populations and other priority steppe bird species (some of them were created ca. 24 years ago). After three little bustard surveys spanning 16 years, we show the designation alone was insufficient to stop a sharp decline. Present management is not sustainable, nor do the designated agri-environmental schemes appear to be enough to benefit the species. The Castro Verde SPA, which holds the most important breeding population, has a specific voluntary agri-environmental program based on livestock production with a significant area contracted to benefit grassland birds (about 30% of the SPA area), but it was nonetheless ineffective in halting population loss of little bustards, that declined 40% in just six years (Table S1).

Overall, we argue that agri-environmental measures have been poorly designed, not including adequate measures to ensure proper breeding habitat for the species. Additionally, despite extensive knowledge of the rate of decline and what is causing it for over 7 years, no additional management measures have been implemented^7,9^. The species lacks a national conservation strategy and effective habitat management. Emergency actions need to take place to implement effective conservative management schemes that ensure optimal breeding habitat for both breeding males and females^8,15^.

## Supplementary Information

Supplementary Information.

## Data Availability

The data matrix used for the analysis will be made available in Dryad upon manuscript acceptance for publication (doi: https://doi.org/10.5061/dryad.7sqv9s4xc).
