# Supplementary material for: A nationwide collapse of a priority grassland bird related to livestock conversion and intensification
Source: Sci Rep. 2023 Jun 29;13:10005. doi: 10.1038/s41598-023-36751-8 (PMC10310727; doi:10.1038/s41598-023-36751-8)
Supplement: Supplementary file 1 — Supplementary Information. [file 41598_2023_36751_MOESM1_ESM.docx]

# Supplementary Material

**A nationwide collapse of a priority grassland bird related to livestock conversion and intensification**

João Paulo Silva, Ana Teresa Marques, Carlos Carrapato, Rui Machado, Rita Alcazar, Ana Delgado, Carlos Godinho, Gonçalo Elias, João Gameiro


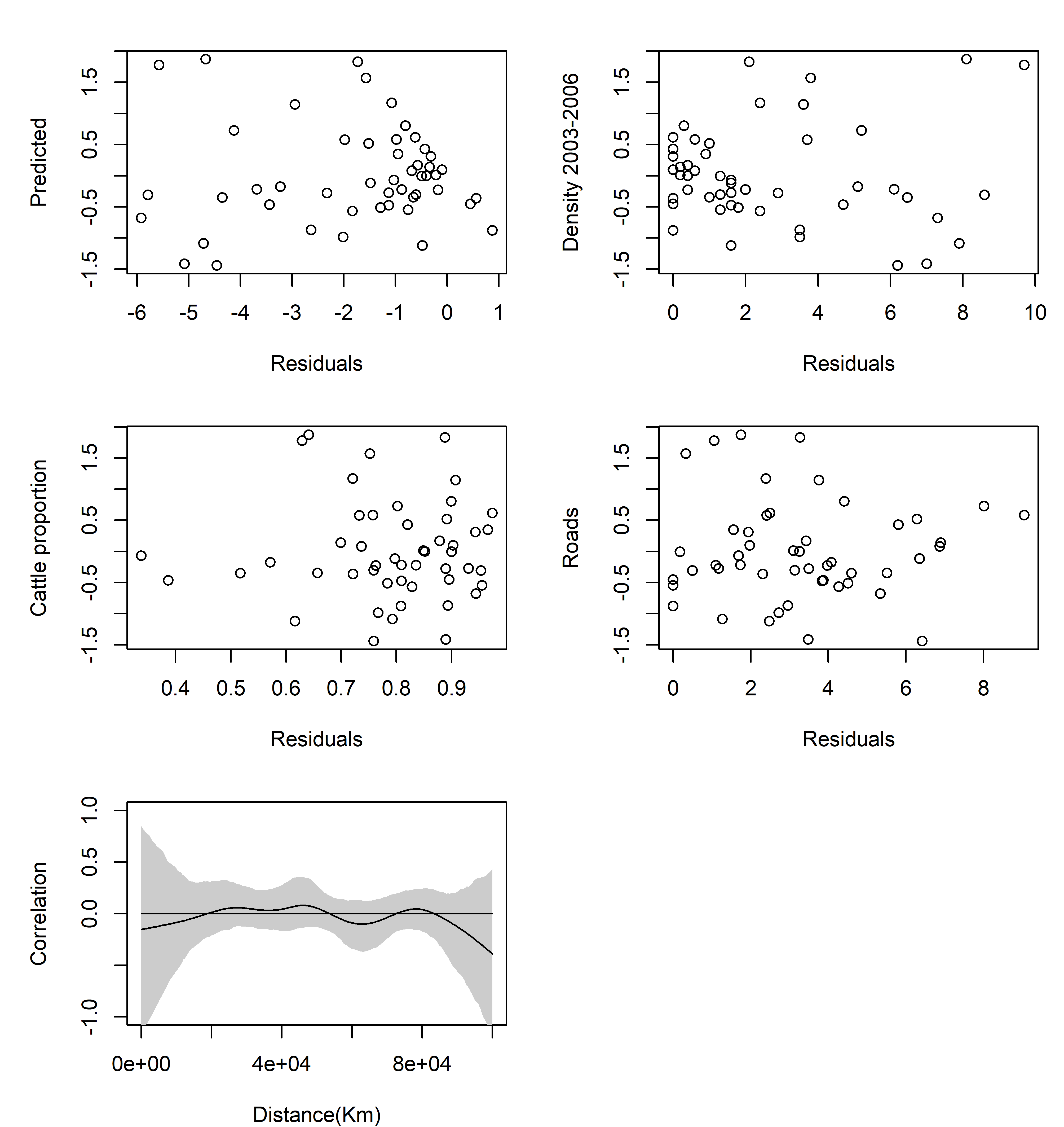


Figure S1 – Validation plots the GAM model: residuals versus fitted values and residuals versus explanatory variables. A Spline correlogram describing the spatial autocorrelation in the residuals is presented in the bottom right corner. Lines represent the estimate (in the middle) and the 95% confidence envelopes (grey shadow) using 1000 bootstrap resamples.

Table S1: Mean, minimum (min) and maximum (max) male density (birds/100ha) and male estimates for each of the Special Protection Areas (SPAs) surveyed for the three little bustard national surveys in continental Portugal. Difference in male estimates and in proportion of population is also presented, and overall trend from the first to the last surveys (considering stable populations with variations up to 10%) are also presented.

|  |  | 2003-2006 | | | | | | 2016 | | | | | | 2022 | | | | | | Variation | | | | | |  |
| --- | --- | --- | --- | --- | --- | --- | --- | --- | --- | --- | --- | --- | --- | --- | --- | --- | --- | --- | --- | --- | --- | --- | --- | --- | --- | --- |
|  |  | density | | | estimate | | | density | | | estimate | | | density | | | estimate | | | 2003/06 - 2016 | | 2016 - 2022 | | 2003/06 - 2022 | | Overall trend |
| SPA | Area (ha) | mean | min | max | mean | min | max | mean | min | max | mean | min | max | mean | min | max | mean | min | max | # | % | # | % | # | % |  |
| Campo Maior | 9 575 | 3.6 | 2.1 | 5.0 | 204 | 122 | 285 | 1.0 | 0.0 | 2.0 | 55 | 0 | 115 | 1.8 | 0.5 | 3.0 | 111 | 33 | 188 | -149 | -73.0 | 56 | 101.0 | -93 | -45.8 | **↓** |
| Castro Verde | 79 066 | 5.8 | 3.8 | 7.8 | 3390 | 2237 | 4544 | 5.3 | 3.3 | 7.3 | 3090 | 1966 | 4222 | 3.2 | 1.6 | 4.8 | 1847 | 942 | 2752 | -300 | -8.8 | -1243 | -40.2 | -1543 | -45.5 | **↓** |
| Cuba | 5 049 | 8.1 | 6.1 | 10.2 | 350 | 260 | 439 | 8.2 | 5.3 | 11.1 | 353 | 228 | 477 | 5.3 | 2.9 | 7.6 | 227 | 127 | 327 | 3 | 0.9 | -126 | -35.7 | -123 | -35.2 | **↓** |
| Évora | 53 134 | 1.1 | 0.2 | 2.0 | 394 | 86 | 703 | 0.7 | 0.1 | 2.8 | 247 | 17 | 925 | 0.2 | 0.0 | 0.4 | 53 | 0 | 124 | -147 | -37.3 | -194 | -78.6 | -341 | -86.6 | **↓** |
| Monforte | 1 593 | 1.1 | 0.2 | 2.0 | 22 | 4 | 41 | 1.0 | 0.0 | 2.2 | 20 | 0 | 43 | 0.9 | 0.1 | 1.7 | 18 | 2 | 34 | -2 | -9.1 | -2 | -11.4 | -4 | -19.5 | **↓** |
| Moura/Mourão/Barrancos | 89 825 | 3.8 | 1.2 | 6.3 | 828 | 293 | 1364 | 0.6 | 0.0 | 1.2 | 188 | 0 | 384 | 0.5 | 0.0 | 1.3 | 149 | 0 | 367 | -640 | -77.3 | -39 | -20.7 | -679 | -82.0 | **↓** |
| Piçarras | 2 827 |  |  |  |  |  |  | 2.5 | 0.0 | 5.2 | 47 | 0 | 99 | 2.1 | 0.8 | 3.5 | 60 | 22 | 99 | -75 | -61.5 | 13 | 28.5 | -62 | -50.5 | **↓** |
| Reguengos | 8 141 | 4.4 | 2.8 | 5.9 | 252 | 164 | 340 | 2.2 | 0.9 | 3.6 | 112 | 45 | 179 | 1.8 | 0.7 | 2.9 | 94 | 36 | 151 | -140 | -55.6 | -18 | -16.2 | -158 | -62.8 | **↓** |
| São Vicente | 3 712 | 8.6 | 5.9 | 11.3 | 318 | 219 | 418 | 2.9 | 1.1 | 4.7 | 107 | 39 | 175 | 2.5 | 0.9 | 4.0 | 91 | 34 | 149 | -211 | -66.4 | -16 | -14.7 | -227 | -71.3 | **↓** |
| Torre da Bolsa | 2 722 | 7.3 | 3.6 | 11.0 | 217 | 108 | 326 | 0.4 | 0.0 | 1.0 | 12 | 0 | 28 | 0.7 | 0.0 | 1.5 | 20 | 0 | 41 | -205 | -94.5 | 8 | 65.0 | -197 | -90.9 | **↓** |
| Vale do Guadiana | 76 578 | 2.7 | 1.3 | 4.1 | 494 | 207 | 638 | 3.5 | 1.7 | 5.2 | 640 | 312 | 969 | 1.4 | 0.5 | 2.3 | 298 | 100 | 495 | 146 | 29.6 | -342 | -53.4 | -196 | -39.7 | **↓** |
| Vila Fernando | 5 261 | 6.1 | 3.7 | 8.4 | 285 | 174 | 397 | 3.0 | 1.7 | 4.3 | 140 | 78 | 202 | 2.2 | 1.1 | 3.4 | 105 | 51 | 159 | -145 | -50.9 | -35 | -25.1 | -180 | -63.2 | **↓** |
| Veiros | 1 960 | 3.6 | 1.7 | 5.6 | 66 | 31 | 101 | 2.4 | 0.9 | 4.0 | 44 | 16 | 72 | 2.3 | 0.6 | 4.0 | 42 | 11 | 73 | -22 | -33.3 | -2 | -4.6 | -24 | -36.4 | **↓** |
| Non-SPA IBA |  |  |  |  |  |  |  |  |  |  |  |  |  |  |  |  |  |  |  |  |  |  |  |  |  |  |
| São Pedro Solis | 14 314 | 6.5 | 5.0 | 7.9 | 647 | 503 | 791 | 2.4 | 1.0 | 3.8 | 224 | 91 | 356 | 1.7 | 0.5 | 3.0 | 194 | 54 | 335 | -423 | -34.6 | -29 | -13.1 | -453 | -70.0 | **↓** |
| Alter do Chão | 2 000 | 2.1 | 1.1 | 3.2 | 43 | 21 | 64 | 2.7 | 1.0 | 4.3 | 80 | 30 | 129 | 2.4 | 0.7 | 4.2 | 49 | 13 | 84 | 37 | 86.4 | -31 | -39.2 | 6 | 13.4 | **↑** |
